# The BSR-PsA: study protocol for the British Society for Rheumatology psoriatic arthritis register

**DOI:** 10.1186/s41927-021-00189-0

**Published:** 2021-05-17

**Authors:** Gareth T. Jones, Gary J. Macfarlane, Karen Forrest Keenan, Paul McNamee, Aileen R. Neilson, Stefan Siebert, A. David Burden, Lesley Kay, Philip S. Helliwell

**Affiliations:** 1 Epidemiology Group, Aberdeen Centre for Arthritis and Musculoskeletal Health, University of Aberdeen, Health Sciences Building, 1st floor, Foresterhill, Aberdeen, AB25 2ZD UK; 2 Health Economics Research Unit, School of Medicine, Medical Sciences and Nutrition, University of Aberdeen, Aberdeen, UK; 3 Usher Institute, Edinburgh Medical School: Molecular, Genetic and Population Health Sciences, University of Edinburgh, Edinburgh, UK; 4 Institute of Infection, Immunity and Inflammation, University of Glasgow, Glasgow, UK; 5 Musculoskeletal Services Directorate, The Newcastle upon Tyne Hospitals NHS Foundation Trust, Newcastle, UK; 6 Leeds Institute of Rheumatic and Musculoskeletal Medicine, University of Leeds, Leeds, UK

**Keywords:** Psoriatic arthritis, Targeted therapy, Biologics, Biosimilars, Registry, Real world evidence

## Abstract

**Background:**

Psoriatic arthritis (PsA) presents a unique clinical challenge. Affecting joints, skin, nails, and other organs, it is associated with various comorbidities and has a significant impact on quality of life, social participation and working life. While biologic and other targeted synthetic disease modifying anti-rheumatic drugs (bDMARDs and tsDMARDs) have revolutionised therapy, questions remain about the long-term safety of these agents, and their effectiveness and cost-effectiveness in the real-world clinical setting.

**Methods/design:**

The British Society for Rheumatology Psoriatic Arthritis Register (BSR-PsA) is a prospective registry of patients with PsA, recruited from across Great Britain, who are (a) commencing a bDMARD/tsDMARD; or (b) naïve to all bDMARDs/tsDMARDs. Ethical approval was given by the NHS West of Scotland Research Ethics Committee 3 (reference: 18/WS/0126). Clinical data are extracted from participants’ medical records, including symptom onset and diagnosis, joint, skin and nail symptoms, dactylitis and enthesitis. Physical measurements (height, weight and 66/68 joint counts) and a detailed drug history are taken. Participants are also asked to complete questionnaires comprising instruments relating to general health and quality of life, axial disease, sleep and fatigue, impact of disease, functional status, mental health, other symptoms, and occupational status. The study duration is 5 years in the first instance, and all participants are followed up annually until the end of the study. Participants commencing a bDMARD/tsDMARD are also followed up three and six months after the start of therapy. Disease activity, including C-reactive protein, is assessed at each visit; and participants from some centres are invited to donate blood and urine samples for the creation of a biobank.

**Discussion:**

Complementing data from randomised trials, results from this study will contribute to the evidence base underpinning the clinical management of psoriatic arthritis. Various analyses will determine the effectiveness and safety of bDMARDs/tsDMARDs in the real-world, will examine the clinical and biological predictors of treatment response, and will provide real-world data on the cost-effectiveness of these therapies, as well as providing informative data important to patients such as quality of life and occupational outcomes.

**Trial registration:**

The full study protocol is registered on the Open Science Framework (https://osf.io/jzs8n).

## Background

Psoriatic arthritis (PsA) is an inflammatory arthritis characterised by synovitis, enthesitis, osteitis, and skin and nail disease. There are several phenotypes, including oligoarticular disease, affecting only a few joints, often unilaterally; polyarticular disease, affecting five or more joints, bilaterally; and the severe and destructive, but less common, arthritis mutilans. It has a significant impact on quality of life, social participation and working life and is associated with an increased cardiovascular risk, likelihood of metabolic syndrome, and other important physical and psychological comorbidities.

The advent of TNF inhibition significantly improved outcomes for patients with PsA, and anti-TNF therapy is now established in the management of PsA [1–5]. More recently, other important targets have been identified and therapies have developed apace. Accordingly, in addition to TNF inhibitors, the rheumatologist now has access to agents that inhibit the IL-12/23, IL-17, JAK and PDE4 pathways, some of which have already demonstrated impressive results in other rheumatological diseases and/or psoriasis.

For many therapeutic agents, good quality long-term data on effectiveness, cost-effectiveness, safety, and other important outcomes in PsA are limited, and much of what is known about the performance and safety of these agents comes from clinical trials. Although these data are informative, it is often collected on a narrow subset of patients and we have shown, in axial spondyloarthritis, treatment response to biologic therapy in ‘real-world’ patients is significantly lower than is reported in clinical trials [6]. In PsA, observational ‘real-world’ evidence is needed, to complement data from randomised trials. This should seek to determine the effectiveness of treatment on all aspects of phenotype, and indeed whether treatment effectiveness varies with disease phenotype. Similarly, it is important to determine the safety and effectiveness of therapies among patients with the whole range of comorbidities seen in clinical practice, especially those that would result in ineligibility from clinical trials. Such data would allow the identification of sub-groups of patients who may have a superior treatment response, with participant numbers seldom available in randomised studies. It would also create the environment to study predictors of treatment response that might inform future stratified approaches to patient management.

All pharmacological interventions may be associated with adverse side effects. Of particular relevance, immunosuppressive therapy is considered a potential risk factor for both malignancy and life-threatening infection – although, in clinical practice, these small risks are accepted if the potential patient benefit is proportionately greater. Adverse events occurring frequently, and early during therapy, will be ascertained during phase 2 and 3 clinical trials. However, longer-term complications such as malignancy, rare infections, and indeed all rare events, are unlikely to be detected until large numbers of patients have been treated and followed for a reasonable length of time. Although much can be learned about drug safety from other disease areas, direct extrapolation from one disease to another may not be appropriate, particularly where comorbidities vary between diseases. Also, while real-world data on TNF antagonists has been available for around 20 years, much less is known about newer therapies: the IL-12/23, IL-17A, JAK and PDE4 inhibitors. Informed prescribing of new agents (and arguably new versions of old agents) requires knowledge of the magnitude of risk of adverse events in the longer-term.

Here, we describe the protocol for the British Society for Rheumatology Psoriatic Arthritis Register (BSR-PsA), a multi-centre registry facilitating research on patients with PsA, in secondary care in the Great Britain, who are commencing biologic or other targeted synthetic disease modifying anti-rheumatic drugs (bDMARDs and tsDMARDs), plus a comparison cohort of patients naïve to bDMARD / tsDMARD therapy. It aims to provide high quality long-term data on effectiveness, cost-effectiveness, safety, and the impact of PsA on the individual, including function, quality of life and social (e.g. work) outcomes in the medium- to long-term. It also collects the relevant data to allow robust health economic evaluation and post-marketing surveillance of biologic and other targeted therapies for a period beyond that typically monitored in clinical trials.

## Methods/design

### Study design, population, and procedures

The BSR-PsA is a multi-centre registry of persons aged ≥16 yrs with a clinical diagnosis of PsA and who meet the CASPAR classification criteria for PsA [7]. They are also required to be either:

- Commencing a biologic, biosimilar or targeted synthetic disease modifying anti-rheumatic drug (bDMARD or tsDMARD) approved for the treatment of PsA in the United Kingdom or part thereof, having never previously received that particular agent; or
- Naïve to all bDMARD and tsDMARD products.

Thus, the registry comprises two cohorts, considered ‘exposed’ and ‘non-exposed’ respectively. Participants are excluded if they are unable to communicate in English; are managed in paediatric rheumatology services; are remaining on, or restarting, a bDMARD or tsDMARD that they have previously received; or are unable to give informed consent. The registry inclusion / exclusion criteria are shown in Table 1.

**Table 1:** Registry inclusion / exclusion criteria

Inclusion criteria	Exclusion criteria
**• Patients must …**	**• Patients must not be …**
**•** Have a clinical diagnosis of PsA;	Unable to communicate in English;
**•** Meet CASPAR classification criteria for PsA [7];	Deemed, in any other way, unable to give informed consent;
**•** Be aged ≥16 years;	Managed in paediatric rheumatology services;
**•** Be either:	Currently taking a bDMARD / tsDMARD and not changing treatment;
(a) Naïve to all bDMARD / tsDMARD agents; or	Restarting a specific bDMARD / tsDMARD agent that they have received previously^a^.
(b) Starting a bDMARD / tsDMARD agent, approved for the treatment of PsA in the United Kingdom, having never previously received that particular drug^a^.	

^a^Patients are eligible if they are starting, or switching to, a biosimilar product even if they have previously had (i) the originator product, and/or (ii) a different biosimilar of the same product

Potentially eligible patients with PsA are identified from participating NHS rheumatology departments across the UK. A list of participating centres is available from www.abdn.ac.uk/bsr-psa. Patients are sent a participant information sheet and invitation letter from their usual rheumatology consultant/team ahead of their clinic appointment. Thereafter, at clinic, they are asked whether they wish to participate. Recruitment is supported in England by the National Institute for Health Research (NIHR) Clinical Research Network, and elsewhere in the UK equivalent research infrastructure support is available. Participant recruitment commenced in September 2018 and is currently ongoing.

Participant eligibility is confirmed by the consultant rheumatologist or a suitably qualified member of the local research team with delegated responsibility from the local Principal Investigator. Being an observational study, cohort membership (i.e. whether the participant joins the exposed or non-exposed groups) is not dictated by the study protocol. Rather, it is determined by the treatment decision of the rheumatology consultant, and the patient, as per usual clinical practice.

Patients have at least 24 h to consider the information in the participant information sheet and have the opportunity to ask questions. However, in line with the ethical approval, if they indicate their willingness to participate sooner, the taking of consent is not unduly delayed. Although the majority of recruitment takes place face-to-face, in exceptional circumstances participants may complete the consent form and return it by post, providing that the local research nurse has had an opportunity (either face-to-face or by telephone) to discuss the study with the participant, and to answer any questions.

### Data collection

During the development of the protocol and data collection procedures, a consultation meeting was held with representation from study investigators, patients, and rheumatology consultants, to ensure that data collection was relevant and comprehensive but avoided duplication.

There are three main aspects of data collection.

- Clinical data

Data are recorded on a variety of clinical features, either directly assessed by the research nurse or taken retrospectively from the clinical notes, and includes information regarding:

- Symptom onset and diagnosis;
- Joint disease;
- Skin and nail disease;
- Dactylitis;
- Enthesitis; and
- Comorbidities.

Training was provided for research nurses prior to commencement of the study. Various physical measurements and laboratory test results are also taken. A full list of clinical data collection items is shown in Table 2. In addition, during follow-up, clinical staff are asked to report any significant morbid events as well as being asked to verify any such events notified in patient diaries. Safety reporting is discussed below.

(b) Patient-reported data

**Table 2:** Clinical data collection

Domain	Items
General information	Date of birth; gender; ethnicity; family history of PsA, psoriasis, and other relevant conditions.
Symptom onset and diagnosis	Age at onset for joint disease, and skin disease, separately. Year that patient was first seen in rheumatology.
Joint disease	66/68 joint count.
Skin disease severity/phenotype	Personal history of psoriasis. Current psoriasis: (a) phenotype: plaque, pustular (sub classified as palmoplantar pustulosis or generalised pustular psoriasis); and (b) severity: body surface area affected, static physician global assessment.
Nail disease	Presence of nail disease: nail matrix (e.g. pitting); nail bed (e.g. onycholysis); hyperkeratosis.
Dactylitis	Current swelling of an entire digit; count of involved digits; history of dactylitis.
Enthesopathy	Leeds Enthesitis Index [8].
Physical measurements	Height; weight; waist circumference; and blood pressure.
Comorbidities	Diabetes; ischaemic heart disease; hypertension; hyperlipidaemia; statin use; fatty liver disease; history of malignancy; history of serious infection (infection resulting in hospitalisation, intravenous antibiotics); history of vasculitis; mental health.
Novel therapy exposure	Past history of all bDMARDs and tsDMARDs, including start date, dose, stop date and reason for discontinuation.
Other current and recent therapies	History of conventional synthetic DMARDs, steroids, NSAIDs, PUVA, and systemic therapies for psoriasis. Data recorded will comprise ever received therapy (yes/no); and, if applicable, calendar year. A recent history (past 6 months) will also be taken, comprising start date, dose, and if applicable, stop date and reason for discontinuation. Information will also be collected on any relevant concomitant therapies for comorbidities.
Spinal involvement/imaging	Evidence of (a) juxta-articular new bone formation; (b) any erosion; and/or (c) sacroiliitis, by plain x-ray or MRI. (NB: No new images will be taken specifically for the BSR-PsA.)
Laboratory markers	Recent measure of inflammation (C-reactive protein) from the date of clinic visit date ±4 weeks. Baseline only: rheumatoid factor and anti-citrullinated protein (anti-CCP) antibody, if available.

At baseline and each follow-up point participants are asked to complete a questionnaire. A full list of questionnaire instruments is shown in Table 3 and includes questions on:

- General health;
- Functional status and impact of disease;
- Axial disease;
- Sleep and fatigue;
- Fibromyalgia;
- Mental health;
- Quality of life; and
- Employment status.

**Table 3:** Patient-reported data collection

Domain	Items
General information	Date of birth; education; smoking status, alcohol consumption; and physical activity.
General health	PROMIS Scale v1.2 – Global Health [9]; and GRAPPA/OMERACT visual analogue scales (global, skin and joints) [10].
Axial disease	Bath Ankylosing Spondylitis Disease Activity Index (BASDAI) [11].
Sleep, Fatigue and Fibromyalgia	Jenkins Sleep Scale [12]; PROMIS Short Form – Fatigue 8a [9]; a general question on fatigue for comparability with other studies (have you had problems with fatigue for more than 3 months?); plus, the 2011 modification of the ACR preliminary diagnostic criteria for fibromyalgia [13].
Quality of life, and Impact of disease	Generic instrument (EQ-5D-5L) [14]; plus, the PsA Quality of Life (PsAQoL) [15]; Dermatology Quality of Life Index (DLQI) [16]; and Psoriatic Arthritis Impact of Disease (PsAID) questionnaire [17].
Functional status	Health Assessment Questionnaire (HAQ) [18].
Mental health	PROMIS Short Form – Anxiety 4a and Depression 4a questionnaires [9] plus, for comparability with other studies, a question on anhedonia taken from the PHQ-9 [19].
Employment status	Employment status, and the impact of PsA on employment: Work Productivity and Activity Impairment Questionnaire: Specific Health Problem (WPAI: SHP) [20].

At baseline, participants are also asked for their preference – postal, or online – which determines the approach for follow-up questionnaires.

(iii) Record linkage

Participants are asked for their consent to link their study data to records held by NHS Digital (www.digital.nhs.uk) for surveillance and notification of any malignancy and/or mortality. In addition, for persons recruited in Scotland data are linked to the Scottish Morbidity Records held by National Health Services Scotland (www.ndc.scot.nhs.uk), providing information on inpatient episodes (including day case) and outpatient attendances.

### Follow-up

The study duration is 5 years in the first instance, and all participants are followed up annually until the end of the study, unless they withdraw. In addition, patients commencing or switching to bDMARD or tsDMARD therapy (either at recruitment, or during the course of the study) will be followed up three and six months after the start of therapy. Ordinarily, recruiting centres endeavour to ensure that each follow-up visit takes place within ±28 days of the required time.^1^ Each follow-up visit involves the collection of clinical and patient-reported data, as per baseline. To decrease the burden for recruiting centres, follow-up data collection is designed to collect clinical data using a schedule that maps to usual clinical practice insofar as possible, although data collection can also take place at additional study-specific visits.

During follow-up participants are asked to keep diaries to record any hospital admissions, new drugs prescribed, new referrals and/or pregnancy. In female participants, in the event of a pregnancy, a supplementary questionnaire is sent, shortly after the expected date of delivery, to collect information about the pregnancy, and pregnancy outcome. If a pregnancy is reported in a study participant's partner, the questionnaire is sent to the participant to pass on to their partner, with a separate participant information sheet and consent form.

### Health economics

Health care costs are collected from three main sources: clinical records, patient diaries, and record linkage. Data is collected on the frequency and type of NHS resource use, such as hospital service use related to PsA and reflects the type and duration of inpatient stays, and the type and frequency of outpatient visits. Costs include the resource use associated with usual management of the condition, together with costs associated with treatment for serious adverse events. Quality of life data will be collected from patient questionnaires, determined by the EQ-5D-5L, which enables direct calculation of quality-adjusted life years (QALYs).

### Safety

Serious adverse events are defined as per the NIHR Clinical Trials Toolkit (www.ct-toolkit.ac.uk/glossary), i.e. any event that results in death, is life threatening, requires hospitalisation or prolongation of existing hospitalisation, results in persistent or significant disability or incapacity, or is a congenital anomaly or birth defect. In addition, information on various events of special interest is collected. These events, listed in Table 4, while not necessarily constituting serious adverse events are of particular clinical interest and warrant additional data collection.

**Table 4:** Events of special interest

Event of special interest	Further explanation
Aplastic anaemia, pancytopenia, serious neutropenia	–
Cerebrovascular accident	Stroke and/or transient ischaemic attack.
Demyelination, optic neuritis	–
Mental health	≥1 new prescription for depressive illness and/or ≥ 1 new prescription for anxiety/nervousness.
Hepatitis B reactivation	–
Hypersensitivity reaction	Including skin eruptions and rashes, oedemas, anaphylactic reactions, and non-specific hypersensitivity – e.g. Stevens Johnson syndrome, erythema multiforme, toxic epidermal necrosis. Life-threatening anaphylactoid reactions including effects on blood pressure and respiratory effects as defined for anaphylaxis [21].
Inflammatory bowel disease	Worsening of existing condition, or new onset.
Lymphoproliferative malignancy	–
Malignancy, skin cancer, Bowen’s disease	Including solid tumours, haematological tumours, melanoma, and non-melanoma skin cancers.
Major adverse cardiovascular events	Including all incident myocardial infarctions and acute coronary syndrome. Sudden cardiac death, death due to myocardial infarction, heart failure, stroke and other cardiovascular causes; myocardial infarction and non-fatal stroke.
Pregnancy	–
Psoriasis flare	Worsening of existing psoriasis, or paradoxical psoriasis onset.
Pulmonary embolism	–
Serious congestive heart failure	–
Infection	Serious infection – i.e. infection resulting in death, or is life threatening and/or requiring hospitalisation and/or intravenous antibiotics. Systemic candida infection, and all recurrent candida and other fungal infections.
Serious hepatic dysfunction or failure	–
Serious lower GI ulcer, bleed or perforation	–
Serious lupus, or lupus-like illness	–
Serious haemorrhage	–
Suicide behaviours	Including suicide ideation, suicide attempt, and suicide.
Tachyarrhythmia	Including atrial fibrillation and flutter, supraventricular tachyarrhythmia and tachycardia, and ventricular tachycardia, fibrillation, and flutter.
Tuberculosis	–
Vasculitis	Including renal and nodular vasculitis, polyarteritis nodosa, temporal arteritis, and all other vasculitis conditions.

### Biobank

Recent evidence suggests that different PsA phenotypes may be determined by distinct genotypes and cytokine responses [22]. This gives rise to specific testable hypotheses about treatment response among patients receiving therapeutic agents that ostensibly target single pathways. Not available from the start of the study, but beginning in 2021, we will collect and store urine, serum, and plasma from a sub-sample of participants to create a PsA biobank which will facilitate analyses at the genomic, epigenetic, transcriptomic (including non-coding RNA species) and metabolomics level.

Depending on local facilities, participants commencing bsDMARD or tsDMARD therapy (either at recruitment, or during follow-up) will be given the option of contributing to a biobank. Those who consent will be asked to give blood and urine samples at their normal clinical visit when commencing therapy, and at three-month follow-up. Blood samples will be centrifuged in local facilities and plasma and serum extracted, and samples will be transferred to the NHS Grampian Biorepository where they will be stored for up to 10 years.

### Statistics and data analysis

The study is designed to answer a series of questions related to natural history, outcome, treatment effectiveness and co-morbidities and is therefore not powered on one single analysis. Thus, the following are provided to give an indicative example of study power. Available data suggest that baseline risk of serious infection (infection resulting in hospitalisation, intra-venous antibiotics, or death) is around 20 events per 1000 person-years. To detect a doubling in risk among PsA patients in the exposed versus non-exposed groups we would require 1626 person-years of follow-up in each group. This would provide 90% power at 5% significance and thus, assuming a one-to-one ratio of patients, would require 723 PsA patients per group, each followed-up for an average of 3 years.

The likelihood of achieving a positive treatment response varies between products, and between outcome measures. Based on three commonly used outcomes (the ACR 20/50/70) and three commonly used agents (etanercept/infliximab/adalimumab) the average treatment response is approximately 40% [23]. Thus, assuming the probability of achieving a positive treatment response is 0.40, a sample of this size would have 90% power to detect a 50% increase in the probability of positive treatment response associated with any exposure occurring with a prevalence of 10%.

Initial analyses will consist of comparisons in baseline characteristics between exposed and non-exposed groups. Thereafter, because the study is designed to be able to answer a number of different research questions, the precise analysis will depend on the specific question being asked. All analyses will require a detailed analysis plan to be specified in advance. As indicative example: differences in risk of serious infection between groups will be examined using Cox-proportional hazards regression, allowing for between-group differences as potential confounders and effect modifiers, and results will be expressed as hazard ratios. Depending on the incidence of the outcome events, more sophisticated models may be possible – e.g. allowing the examination of time-varying and/or age-specific risks. In addition, different assumptions will be tested concerning specific risk windows. Treatment success would be pre-specified – e.g. achieving minimal disease activity twelve months after commencing treatment [24] – and initial differences in treatment outcome between exposed and non-exposed groups would be examined with simple descriptive statistics. Thereafter, a logistic model would be fitted to estimate the odds ratio for minimal disease activity between exposed/non-exposed patients. The model would be statistically adjusted for other exposures and patient characteristics associated with both the primary exposure and the outcome, to control for potential confounding.

The study will also provide several health economic outputs: firstly, descriptive statistics in terms of costs and quality of life for the entire registry population – e.g. mean/median costs and EQ-5D-5L values per patient, for first and subsequent years of treatment; and secondly, cost and QALY estimates per patient, controlling for patient differences in disease severity, number and type of adverse events, and other important potential confounding variables. Both costs and quality of life data will be estimated using regression analysis, to control for between patient differences in disease severity, number and type of serious adverse events, and other important confounding variables. Direct estimation of costs and quality of life using large-scale real-world patient data will allow us to produce more estimates of cost-effectiveness. Such results will be able to inform input parameters for future model-based economic evaluations on the costs and effects of all new treatments for PsA in the longer-term.

### Research governance and study oversight

The study was externally peer reviewed as part of the process of applying for funds, competitively, to the British Society for Rheumatology (BSR) and is registered on the Open Science Framework (https://osf.io/jzs8n). Favourable ethical opinion, which applies to all sites, was obtained from the NHS West of Scotland Research Ethics Committee 3 (reference: 18/WS/0126). Appropriate NHS Research and Development approval is obtained prior to the commencement of participant recruitment at each site. The BSR is the legal sponsor for the study, but delegates certain functions to the University of Aberdeen.

The study is co-ordinated by a Study Management Group consisting of the study investigators. A study co-ordinator oversees the study and is accountable to the Chief Investigator. A Data Monitoring Committee is convened by the BSR. The register and data belong to the BSR and oversight of the BSR-PsA is undertaken by the BSR Registers Committee, consisting of BSR members with various other co-opted representatives including the Chief Investigator and Deputy Chief Investigator of the BSR-PsA, patient organisation representatives, and representatives from the BSR’s other registers. Separately, and reporting to the BSR Registers Committee, a Data Monitoring Committee reviews reports of all serious adverse events and events of special interest.

### Dissemination of findings

Dissemination of research output from the BSR-PsA will comply with the policy as specified by the BSR’s Registers Committee. In addition, for output from the main study investigators, lay summaries will be made available online.

## Discussion

Various extant registers across Europe collect some data on PsA, including industry-sponsored and independent registers, but the utility of these data is limited. The BSR Biologics Register for Rheumatoid Arthritis (BSRBR-RA) [25] initially included some patients with PsA although with a predominant focus on rheumatoid arthritis failed to adequately capture all relevant domains in PsA, especially skin disease and information on PsA-relevant outcomes is limited (for example, by using DAS28, rather than full 66/68 joint counts, and no assessment of axial disease. Also, being outside their initial remit, when numbers of patients with rheumatoid arthritis increased, recruitment on PsA ceased. In other registers, such as the British Association of Dermatologists Biologic and Immunomodulators Register (BADBIR) [26], although skin disease is well characterised, the full clinical features of PsA are poorly documented (rheumatological assessment is limited to the single question: Has the patient received a diagnosis by a rheumatologist of psoriatic arthritis?).

There is clear evidence, from randomised trials, that bDMARDs and tsDMARDs are efficacious in the treatment of PsA [23, 27, 28]. However, it has been shown that certain clinical (e.g. oligoarthritis, those with comorbidities) and sociodemographic groups are likely to be under-represented in clinical trial populations [29], and this is likely to introduce potential bias to absolute effect estimates. Indeed, data from the BSR Biologics Register for Ankylosing Spondylitis indicate that in a real-world patient population around half of patients respond to TNF inhibition [6], compared to > 60% in the clinical trials that led to the licensing of these agents. Also, there is some evidence from a large claims database in the USA that the majority of patients commencing TNF or IL-12/23 inhibition had continued their index therapy within a twelve-month period [30]. Long-term studies have the opportunity to examine effectiveness of these agents in real-world clinical populations and allows the examination of patient sub-groups that are seldom available in clinical trials.

The broad inclusion criteria for the exposed group, allowing patients commencing any bDMARD or tsDMARD, is deliberately inclusive to reflect real-world practice. The advent of biosimilars has brought a rapid increase in the number of alternative bDMARDs available to the rheumatologist. Although manufactured to be ‘similar’ to the originator product, and non-inferior in terms of effectiveness and safety profile, biosimilar molecules are not required to be tested in all indications and, subsequently, may be licenced for use in PsA without any clinical trial data in this patient group. Real-world data therefore serve an important function in monitoring effectiveness, safety, and cost-effectiveness of these new products.

Previous economic modelling in PsA, on which management guidelines are based, have employed mapping techniques to estimate quality of life as a function of disability and skin disease [31]. While these indirect methods have some merit, where direct measurement of health utility is lacking, there is some evidence that health utility scores obtained by extrapolating from disease activity and function differ markedly from those calculated directly [32]. Large-scale real-world patient data are required with estimates of QALYs determined using directly collected health economic measures, as well as the clinical tools used elsewhere. This will permit robust estimates of cost-effectiveness and will either challenge or confirm the indirect methods used in economic modelling to date.

In summary, we have described the rationale and design for a prospective PsA registry to provide real-world data with respect to the natural history of PsA and the impact on the individual, including function, work, and quality of life in the medium- to long-term; and the use of effectiveness, cost-effectiveness, and predictors of treatment response of bDMARD and tsDMARD therapeutics. Further, reserving biological samples against an extensive clinical phenotyping database future-proofs the BSR-PsA and facilitates biomarker evaluation and predictive modelling using various mathematical and informatics solutions.

## Data Availability

A copy of the full study protocol, including any future amendments, is available via the Open Science Framework (https://osf.io/jzs8n). Members of the BSR, and external parties, are able to apply to access and analyse BSR-PsA data. Details of how to apply are available from: www.rheumatology.org.uk/practice-quality/registers.

## Abbreviations

bDMARDs: Biologic disease-modifying anti-rheumatic drugs
BSR: British Society for Rheumatology
BSR-PsA: British Society for Rheumatology Psoriatic Arthritis Register
NIHR: National Institute for Health Research
PsA: Psoriatic arthritis
QALYs: Quality-adjusted life years
tsDMARDs: Targeted synthetic disease-modifying anti-rheumatic drugs

## Footnotes

1 Commencing in March 2019, lockdown restrictions during the COVID-19 pandemic meant that all participant recruitment, and almost all follow-up activity, was temporarily put on hold as face-to-face activity was stopped, including non-urgent rheumatology consultations, and research nurses were co-opted back to clinical duties.
